# Skeletal abnormalities detected by SPECT is associated with increased relapse risk in pediatric acute lymphoblastic leukemia

**DOI:** 10.18632/oncotarget.18110

**Published:** 2017-05-23

**Authors:** Fen Zhou, Meiling Zhang, Juan Han, Jinjin Hao, Yan Xiao, Qin Liu, Runming Jin, Heng Mei

**Affiliations:** ^1^ Department of Pediatrics, Union Hospital, Tongji Medical College, Huazhong University of Science and Technology, Wuhan, Hubei, China; ^2^ Institute of Hematology, Union Hospital, Tongji Medical College, Huazhong University of Science and Technology, Wuhan, Hubei, China

**Keywords:** skeletal abnormalities, SPECT, acute lymphoblastic leukemia, relapse

## Abstract

**Objectives:**

Most children with acute lymphoblastic leukemia (ALL) exhibit skeletal abnormalities. This study aimed to investigate bone lesions detected by whole-body bone single-photon emission computed tomography (SPECT) and its prognostic value in children with ALL.

**Methods:**

A retrospective analysis was performed using whole-body bone SPECT scans obtained from children with ALL in our department between June 2008 and June 2012. A total of 166 children newly diagnosed with ALL were included, and the patients were divided into two groups: patients with positive and negative SPECT scans. We compared the clinical characteristics of the two groups and analyzed the relationship between the skeletal abnormalities detected by SPECT and prognosis.

**Results:**

Among the 166 patients, bone scintigraphic abnormalities was detected by SPECT scan in sixty-four patients (38.6%). The most common site was the limbs. There were no significant differences in age, gender, WBC count at diagnosis, risk group and minimal residual disease (MRD) level between SPECT-positive patients and their SPECT-negative counterparts. The event-free and overall survival rates were higher in SPECT-positive patients, but the difference was not statistically significant. However, patients with positive SPECT scans, especially those with multifocal abnormalities (≥3 sites), had a higher rate of relapse (*P* < 0.05). Multivariate analyses identified that abnormal SPECT scan (HR = 3.547, *P* = 0.015) was an independent relapse risk.

**Conclusion:**

Children with ALL and multiple skeletal abnormalities will suffer from relapse. Abnormal SPECT scan was associated with increased relapse risk which might be a potential relapse marker for ALL children.

## INTRODUCTION

Bone pain is both a main and frequently early symptom of leukemia in children; this symptom is related to the large-scale production of hemopoietic tissue in the bone-marrow cavities of long bones and vertebrae [1]. In leukemia patients with bone pain, radiographic examinations, such as X-ray or computed tomography (CT), are usually requested. Radiological abnormalities at diagnosis attributed to the leukemia process have been reported to occur in up to 70% of children. However, the prognostic significance in leukemia of skeletal lesions detected by bone scans remains unclear. Several researchers have denied the presence of any link between bone lesions and survival rate [2-4]. In contrast, other authors have concluded that patients with acute leukemia and skeletal lesions at diagnosis can expect a worse prognosis [5-7].

In this study, we carried out a retrospective research to explore the prognostic value of skeletal abnormalities detected by single-photon emission computed tomography (SPECT) in patients newly diagnosed with acute lymphoblastic leukemia (ALL). We collected the clinical data of children who underwent a whole-body bone SPECT scan, analyzed the relation between bone scintigraphic lesions and clinical characteristics especially the risk of relapse and overall survival.

## MATERIALS AND METHODS

### Patients

We performed a retrospective study of 202 children newly diagnosed with ALL between June 2008 and June 2012. All of the patients were treated in accordance with the CCLG-ALL-2008 Protocol (Children’s Cancer and Leukemia Group) at Wuhan Union Hospital. The diagnoses were established based on morphological, cytogenetic, immunophenotypical and molecular biological assessments. In all, 166 patients, including 110 boys and 56 girls, received a whole-body bone SPECT scan as part of their routine clinical examination. The median age at diagnosis was 6.25 years (range: 13 months to 14 years old). The clinical data were collected anonymously, and the clinical data and SPECT results were analyzed separately and blindly by 2 researchers. The survival data was collected from clinic visit or family contact. This clinical study was approved by the Medical Ethics Committee of Union Hospital, Huazhong University of Science and Technology.

### Whole-body bone SPECT

Whole-body bone SPECT was performed first few days before the time of treatment. All bone scans were obtained using a dual-detector gamma camera with the simultaneous acquisition of anterior and posterior whole-body images. The dose used was 9.3 MBq/kg (250 mCi/kg), with a minimum dose of 110 MBq (3 mCi) and a maximum dose of 750 MBq (20 mCi) 99mTc-MDP. Early and delayed whole-body images were obtained from all patients. Early whole-body images were obtained within 5 min of the injection, while the delayed whole-body images were obtained 2-3 h after injection. The whole-body images including anterior and posterior views were obtained, as well as selected spot views of suspicious areas. All the images were reviewed by two nuclear medicine physicians independently. Any disagreement would be discussed until a consensus was reached. The increased tracer uptake was defined as positive SPECT scan (bone scintigraphic lesions).

### Minimal residual disease measurement

The minimal residual disease (MRD) level was detected at two-time points (TP): TP1, at the end of remission induction around day 33 and TP2, before consolidation therapy (in week 12). Bone marrow aspirates were collected in preservative-free heparin. Leukemia-associated immunophenotypes were determined by multivariable flow cytometry and multiple marker combinations (CD10/CD34/CD45/CD19/CD20/ CD22/CD38, TDT/cCD3/CD45/CD5, CD3/CD8/CD4/CD45 and CD34/CD2/CD45/ CD7) were performed. The sensitivity of MRD is 0.01% (abnormal cells were no less than 1×10^-4^).

### Statistical analysis

The results were either expressed as the mean ± standard deviation or as percentages. The t-test was used to compare two groups of quantitative data. The chi-square test was used to compare two groups of qualitative data. The treatment results were evaluated in terms of event-free survival (EFS) and overall survival (OS). Survival curves were calculated using the Kaplan-Meier method and were compared using the log-rank test. Furthermore, the prognostic value of the abnormal SPECT was identified by the logistic multivariate analyses. *P* values of < 0.05 were considered to be statistically significant. SPSS 13.0 software was used for the statistical analysis.

## RESULTS

### Clinical presentation

Forty-nine (29.5%) of the 166 patients had complaints related to the skeletal system at the time of ALL diagnosis. Forty-six of the patients had bone/joint pain, 1 had septic arthritis-type symptoms (e.g., pain, tumefaction, heat, reduced mobility, and fever), and 2 had a fracture. The skeletal anomalies were localized in the limbs in 13 patients (26%), the breastbone in 2 (4%), the waist in 1 (2%), and the joints in 25 (50%). The affected joints included the knee in 11 (22%), the wrist in 4 (8%), the ankle in 4 (8%), the elbow in 3 (6%) and the coxofemoral in 3 (6%).

### Bone scintigraphic abnormalities

**Figure 1:**
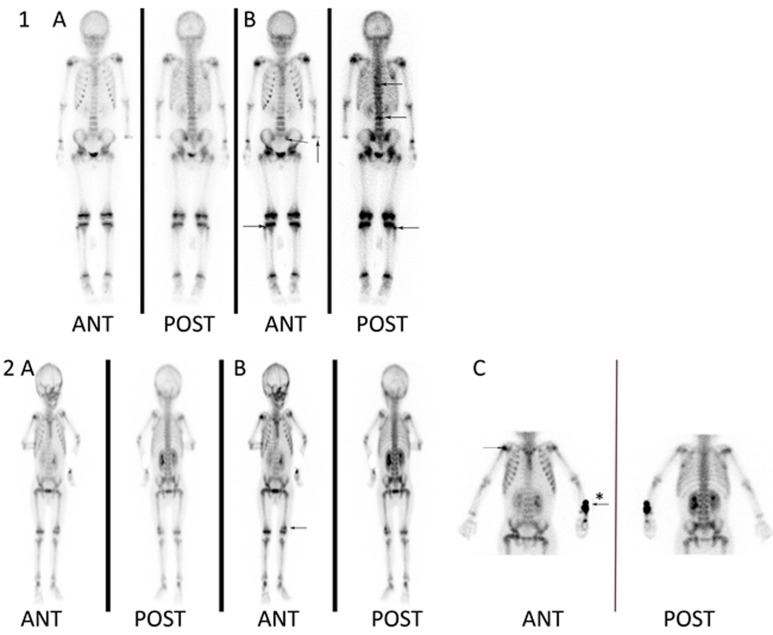
Skeletal abnormalities detected by whole-body bone SPECT scan The increased tracer uptake was defined as skeletal scintigraphic abnormalities. Patient 1 (an 8.5-year-old girl) had no complaints of bone pain. Her whole-body images showed multifocal abnormalities as arrows indicated, including left radius, left side of the sacroiliac joints, right fibula, thoracic vertebra and lumbar vertebra. Patient 2 (a 3.7-year-old boy) was presented with bone pain in his left leg. The whole-body images showed abnormal tracer uptake in the left distal femoral and right shoulder joint. **A.** Early whole-body images were obtained within 5 min of the injection; **B.** delayed whole-body images were obtained 2-3 h after injection; **C.** a high-resolution image of the suspicious area, * the arrow showed the site of superficial vein catheter.

As shown in Figure 1, the whole-body bone images showed increased tracer uptake in several areas, which were defined as positive SPECT scans. Among the 166 patients, sixty-four patients (38.6%) exhibited one or more sites of skeletal abnormalities as detected by SPECT scans. Eighteen patients had more than three sites of bone abnormalities, and 9 patients had more than five sites of bone abnormalities. All of these bone sites included the skull, truncal bones such as the breastbone, ribs, and vertebrae, as well as the sacrum and limbs. The most common site was the limbs (32/64), followed by the vertebrae (24/64) and ribs (23/64). Of vertebrae, 13 thoracic and 11 lumbar vertebrae were showed as bone scintigraphic lesions (Table 1).

**Table 1:** Skeletal involvement in children with ALL detected by SPECT

Site	n (%)
Skull	4 (6.3%)
Breastbone	3 (4.7%)
Clavicle	1 (1.6%)
Scapula	4 (6.3%)
Rib	23 (35.9%)
Spine	24 (37.5%)
Thoracic vertebra	13 (20.3%)
Lumbar vertebra	11 (17.2%)
Pelvis	11 (17.2%)
Limbs	32 (50.0%)
Joint	9 (14.1%)
Sternoclavicular joint	1 (1.6%)
Costovertebral joint	1 (1.6%)
Sacroiliac joint	5 (7.8%)
Shoulder joint	1 (1.6%)
Ankle joint	1 (1.6%)

### Skeletal abnormalities and clinical characteristics

The clinical characteristics of the patients with ALL are shown in Table 2. There were no significant differences in age, gender, white blood cell (WBC) count at diagnosis, risk group or level of TP1 and TP2 MRD between SPECT-positive patients and their SPECT-negative counterparts (chi-square test, *P* > 0.05). While patients with positive SPECT scans had a higher rate of bone pain, with a prevalence of 56.3% (chi-square test, *P* < 0.05), 12.7% of the SPECT-negative patients suffered from bone pain. Of those patients with multifocal skeletal abnormalities (more than 3 sites of bone scintigraphic lesion), most (66.7%) patients had bone pain at diagnosis.

**Table 2:** Clinical features of children with skeletal abnormalities detected by SPECT

	SPECT positive (n=64)		SPECT negative (n=102)	*P* value
Age at diagnosis				
≤10 years	85.9%(55)	90.2%(92)		
>10 years	14.1%(9)	9.8%(10)		0.402
Gender				
Male	67.2%(43)	65.7%(67)		
Female	32.8%(21)	34.3%(35)		0.842
Bone pain	56.3%(36)	12.7%(13)		**0.000**
WBC>50×10^9^/L	7.8%(5)	15.7%(16)		0.137
T lineage	3.1%(2)	6.9%(7)		0.485^b^
BCR/ABL (+)	3.1%(2)	5.9%(6)		0.712^b^
TEL/AML1 (+)	21.9%(14)	11.8%(12)		0.081
Risk group				
Low risk	48.4%(31)	37.3%(38)		0.155
Intermediate risk	34.4%(22)	35.3%(36)		0.904
High risk	17.2%(11)	27.5%(28)		0.129
TP1 MRD^a^ (+)	21.9%(14)	26.5%(27)		0.504
TP2 MRD^a^ (+)	9.4%(6)	8.8%(9)		0.904
Relapse	17.2%(11)	5.9%(6)		**0.019**

^a^MRD: minimal residual disease; TP1: time point 1, at the end of remission induction around day 33; TP2: time point 2, before consolidation therapy ( in week 12). *P* values were determined using the chi-square test and Fisher’s exact test^b^.

In addition, we compared the following biochemical markers related to bone metabolism between SPECT-positive patients and SPECT-negative patients: the serum levels of calcium, magnesium, and phosphate, which are related to bone mineral content; the serum level of alkaline phosphatase (ALP), which is indicative of bone formation; and the ratio of calcium/creatinine, which reflects bone absorption. No differences were found between the two groups (t-test, *P* > 0.05). The blast levels in peripheral blood and bone marrow were also similar in the two groups (t-test, *P* > 0.05) (Supplementary Table 1).

### Skeletal abnormalities and relapse/survival

**Figure 2:**
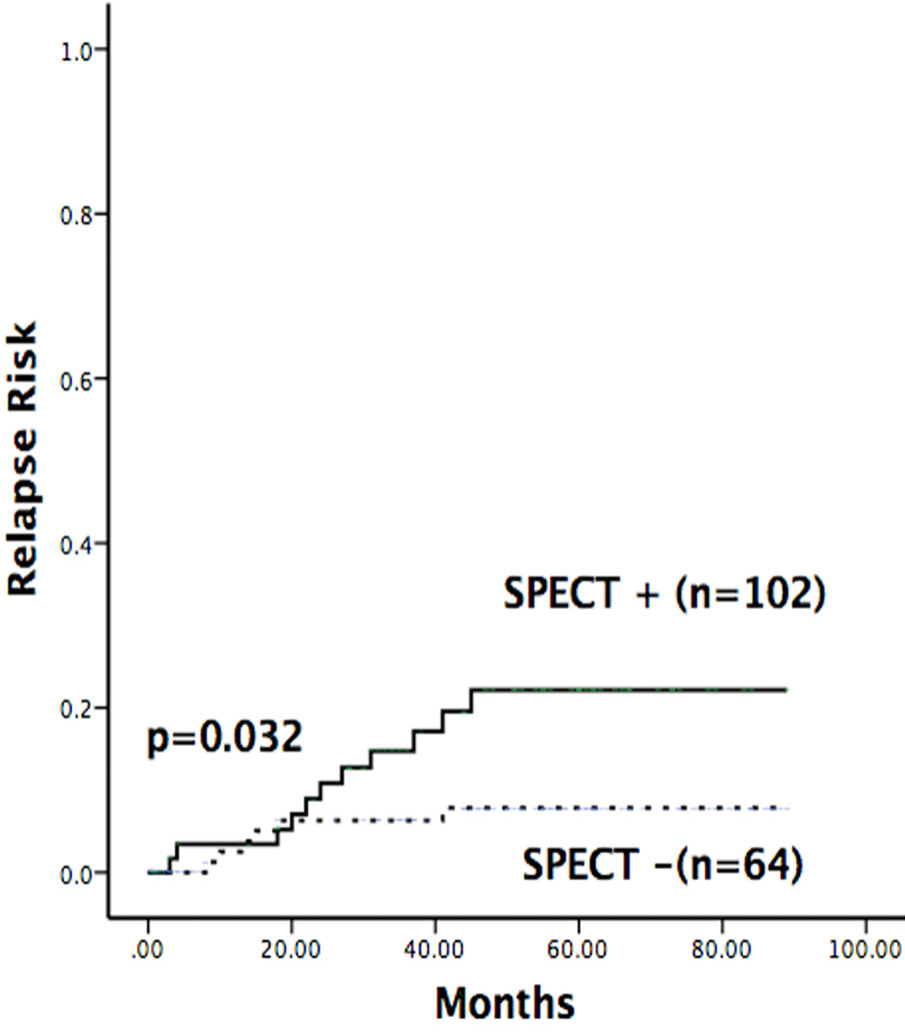
Relapse risk of patients with abnormal SPECT scans The relapse rate of SPECT-positive patients was significantly higher than that of SPECT-negative patients.

**Figure 3:**
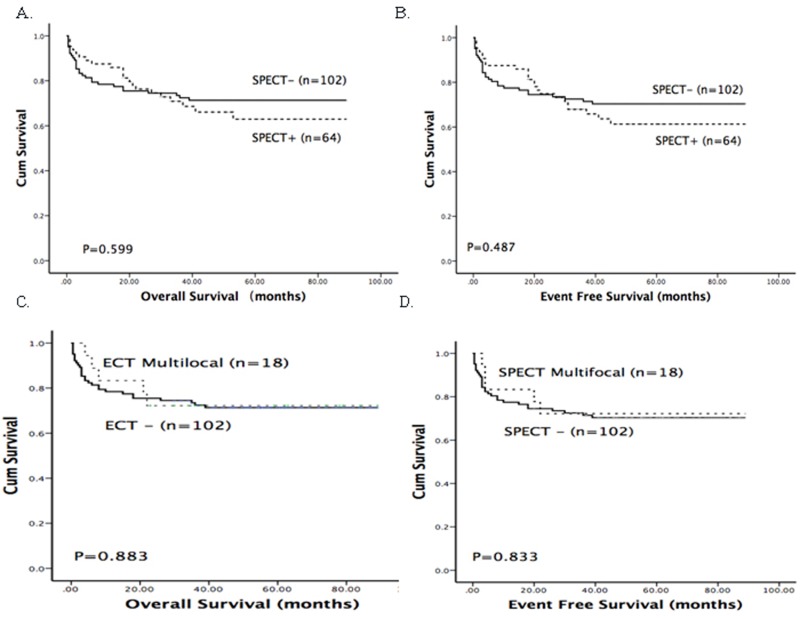
Kaplan-Meier survival curves for overall survival and disease-free survival in a total of 166 ALL patients **A.** and **B.** The OS and DFS of patients with positive (SPECT +) and negative SPECT (SPECT -) results. **C.** and **D.** The OS and DFS of patients with multifocal abnormalities detected by SPECT (SPECT multifocal).

In our study, 17 patients suffered from relapse including three extramedullary relapse (2 CNS and 1 testicular) and 14 isolated bone marrow relapse (Supplementary Table 2). As shown in Table 2, skeletal abnormalities detected by SPECT were demonstrated to be associated with relapse in our study. Of 64 SPECT-positive patients, 17.2% (11/64) suffered from relapse, whereas this was true for only 5.9% (6/102) of patients without SPECT abnormalities (chi-square test, *P* < 0.05). Patients with multifocal abnormalities had a higher risk of relapse; the rate of relapse in this population was 27.8% (vs. 5.9%, *P* < 0.05). The cumulative incidence of relapse was shown in Figure 2. At 3 years, the relapse rate of SPECT-positive patients was 22.2%, which was significantly higher than that of SPECT-negative patients (6.3%) (*P* < 0.05). Cox proportional hazards model was further conducted to identify whether abnormal SPECT scan was an independent prognostic factor for relapse. After multivariate analysis, abnormal SPECT scan (HR = 3.547, *P* = 0.015) and risk group (HR = 3.824, *P* = 0.01) were confirmed as independent markers for ALL relapse (Table 3). However, positive SPECT was not associated with survival. The Kaplan-Meier method and log-rank tests showed that there were no differences of the EFS and OS between SPECT-positive patients and SPECT-negative patients (*P* > 0.05) (Figure 3).

**Table 3:** Univariate and multivariate analysis of factors influencing relapse

Variable	Univariate analysis		Multivariate analysis	
	HR(95%CI)	P value	HR(95%CI)	P value
Gender	0.992(0.367-2.681)	0.987		
Age at diagnosis	2.735(0.888-8.426)	0.08		
Bone pain	1.016(0.358-2.884)	0.976		
White blood cell count	1.119(0.256-4.893)	0.882		
T Lineage	21.686(0.001-373971.584)	0.536		
High risk group	2.962(1.088-8.062)	0.034	3.824(1.373-10.645)	0.01
BCR-ABL (+)	20.709(0.000-4.718*10^9)	0.758		
TP1 MRD	1.368(0.482-3.886)	0.556		
TP2 MRD	21.872(0.002-254144.653)	0.518		
Abnormal SPECT scan	2.839(1.049-7.686)	0.04	3.547(1.284-9.802)	0.015

MRD: minimal residual disease; TP1: time point 1, at the end of remission induction around day 33;TP2: time point 2, before consolidation therapy ( in week 12)

## DISCUSSION

99mTc-MDP SPECT is widely used in cancer patients to detect bone metastasis. The increased tracer uptake on whole-body imaging mostly represents bone infiltration. In this article, we examined the role of skeletal abnormalities detected by SPECT in the prognosis of pediatric ALL. We observed that among ALL children, the risk of relapse was closely related to the abnormal SPECT scan. Patients who had more skeletal lesions at diagnosis had a higher risk of relapse. This observation is important as it provides some evidence that there is a link between bone infiltration and leukemia relapse.

As one potential explanation of our findings, leukemia cells can escape into the bone and periosteum, which may ultimately lead to relapse. This hypothesis was proven by related studies that isolated bone relapse occurs during complete bone marrow remission in childhood ALL. The majority of these patients experienced subsequent relapses after isolated bone recurrence. One child was even still in complete remission for two years after osseous relapse [8-10]. These observations strongly suggest that structural bone may be a sanctuary site for leukemic cells. Radiographic imaging could be utilized to detect skeletal lesions in children with ALL, especially in patients with multiple bone lesions at diagnosis or bone pain during remission. Skeletal abnormalities may serve as markers of leukemia cell infiltration.

Within the field of cancer research, focus on the study of MRD has grown exponentially over the past several years. MRD encompasses circulating tumor cells (CTCs - cancer cells on the move via the circulatory or lymphatic system), disseminated tumor cells (DTCs - cancer cells that have escaped into a distant site), and resistant cancer cells surviving therapy, be they local or distant, all of which may ultimately give rise to local relapse or overt metastasis. The leukemic cells infiltrating bone may be the DTC of ALL patients that cannot be detected by bone marrow MRD but can be killed by the chemotherapy. This can explain why we found no relationship between skeletal abnormalities and the level of MRD.

Our result is partially consistent with some other studies that suggest that initial radiological bone lesions are correlated with the subsequent clinical course. Researchers who studied the prognostic significance of radiological bone involvement in 98 children with ALL found that the mean duration of remission and survival was much shorter in patients with multiple bone lesions (3 or more bones) than in those without bone involvement. No significant difference was found between cases with 1 or 2 bone lesions and cases without bone lesions [6]. Khanna also noted that patients with more radiographic skeletal lesions had a lower survival rate [5].

In our study, skeletal abnormalities influenced the risk of relapse but not overall survival. This may have occurred for several reasons: first, deaths are typically rarer than relapses; therefore, it is more difficult to show differences in overall survival using statistics. Second, relapses are not always equal to deaths, as some children can be saved. At last, a reduction in relapse may be offset by more treatment-related mortality. These findings need to be confirmed by larger studies with longer periods of follow-up.

What also surprised us is that BCR/ABL+ and high levels of MRD showed no correlation with relapse. BCR/ABL+ has been proven to be associated with the poor outcome of ALL, including poor response to chemotherapy and relapse. However, as the usage of tyrosine kinase inhibitors (TKI) such as Imatinib or Dasatinib increases in childhood ALL, the Ph+ patients have a better prognosis. TKIs administered in therapy can dramatically reduce MRD and improve the outcome of childhood Ph+ ALL [11-12]. The incorporation of MRD in risk allocation has markedly improved the ability to determine prognosis early in therapy; however, many patients who relapse are MRD-negative [13]. In our study, there were 13 (72.2%) MRD-negative patients suffering from relapse. Furthermore, in the 41 children positive for MRD, 13 died or abandoned the study, and most of the events occurred during chemotherapy. This may influence the results of the analysis of MRD and relapse.

Compared with X-ray, bone scintigraphy has been shown to be more sensitive in identifying skeletal metastatic lesions [14-15]. In the past, X-rays were used to examine skeletal changes in children with ALL. Dini et al. investigated the whole-body radiography of 119 children with ALL and found that 17.2% of the children had bone changes [16]. Rajantie et al. reported that of 137 children who had long-bone radiographs, 45 (32.8%) had bone lesions [17]. The 166 children in our study had all received whole-body bone scans, and 38.6% had bone abnormalities. Whole-body bone SPECT is widely used in patients with lung or breast cancer to detect bone metastasis. Love et al. demonstrated that approximately 75% of patients with skeletal pain show abnormal uptake on bone scintigraphs and that 25%- 45% of asymptomatic patients with malignancy have bone metastasis [18]. In this paper, 73.5% of patients with bone pain showed positive SPECT results, and approximately 23% of patients without skeletal symptoms also had radiographic skeletal changes.

Of course, the value of SPECT in identifying leukemia infiltration is limited because of the low specificity. Abnormal lesions in bone scans appear as areas of either increased or decreased intensity, which is nonspecific. These findings may have causes other than tumors, such as fractures or degenerative diseases. Occasionally, abnormal findings in the spine are difficult to distinguish on whole-body bone scans. To avoid these shortcomings, SPECT/CT can be used to localize and interpret a lesion correctly and to help differentiate benign and malignant lesions [19-21]. SPECT/CT can also detect 20%-50% more lesions than whole-body bone scans in the spine, which is the most common site of skeletal metastases [22-24].

## CONCLUSIONS

In this study, we investigated skeletal abnormalities in children with ALL using whole-body bone SPECT scans and found that skeletal abnormalities are closely related to relapse. Abnormal SPECT scan might be potential relapse marker for ALL children.

## SUPPLEMENTARY MATERIALS TABLES


