# Supplementary material for: Skeletal abnormalities detected by SPECT is associated with increased relapse risk in pediatric acute lymphoblastic leukemia
Source: Oncotarget. 2017 May 23;8(45):79347–55. doi: 10.18632/oncotarget.18110 (PMC5668046; doi:10.18632/oncotarget.18110)
Supplement: Supplementary file 1 [file oncotarget-08-79347-s001.pdf]

Skeletal abnormalities detected by SPECT is associated with increased relapse risk in pediatric acute lymphoblastic leukemia

Supplementary Materials

Supplementary Table 1: Biochemical measures of children with skeletal abnormalities detected by SPECT

	SPECT positive (n=64)	SPECT negative (n=102)	*P* value
ALP[a] (U/L)	131.08±41.45	147.88±83.20	0.087
Calcium (mmol/L)	2.34±0.16	2.33±0.23	0.812
Magnesium (mmol/L)	0.94±0.14	0.94±0.144	0.604
Phosphate (mmol/L)	1.55±0.08	1.63±0.37	0.102
Creatinine	35.13±14.72	34.67±22.21	0.882
Ca/Cr[b]	0.08±0.027	0.08±0.027	0.588
Blasts of PB[c]	0.32±0.29	0.38±0.34	0 0.263
Blasts of BM[d]	0.80±0.22	0.85±0.13	0.075

[a]ALP: alkaline phosphatase;[b]Ca/Cr: calcium/creatinine; [c]PB: peripheral blood; [d]BM: bone marrow. *P* values were determined by the t-test.

Supplemental Table 2: Clinical characteristics of patients with relapse

No.	Age	Gender	SPECT	Site	Type	TP1 MRD	TP2 MRD
1	3Y2M	M	+	BM	B-LR (TEL/AML1)	-	-
2	3Y10M	M	+	BM	B-LR	-	-
3	4Y9M	M	+	BM	B-LR	-	-
4	9Y	M	+	TL	B-LR	-	-
5	10Y1M	F	+	BM	B-IR	0.1%	-
6	4Y1M	M	+	BM	B-IR	-	-
7	8Y8M	M	+	BM	B-HR	0.27%	-
8	12Y7M	M	+	BM	B-IR	-	-
9	2Y	F	+	BM	B-LR	-	-
10	1Y5M	M	+	BM	B-LR	-	-
11	2Y5M	M	+	BM	B-HR	9%	-
12	10Y9M	M	-	CNSL	B-HR	-	-
13	3Y5M	M	-	BM	B-HR	-	-
14	11Y3M	F	-	BM	B-IR	-	-
15	8Y10M	F	-	BM	B-HR (MLL/AF4)	1.03%	-
16	5Y4M	F	-	CNSL	B-HR	-	-
17	5Y7M	F	-	BM	B-IR (TEL/AML1)	0.1%	-

BM: bone marrow; TL: testicular leukemia; CNSL: central nervous system leukemia; LR: low risk; IR: intermedium risk; HR: high risk; TP1: time point1, at the end of remission induction around day 33 and TP2, time point 2, before consolidation therapy (in week 12).
